# A study on the effects of language and visual art integrated teaching on language learning performance and satisfaction of ethnic minority students in China

**DOI:** 10.3389/fpsyg.2022.1048635

**Published:** 2022-11-11

**Authors:** Yao Zhang, Simeng Jia

**Affiliations:** ^1^School of Economics, Minzu University of China, Beijing, China; ^2^School of Management, Hebei Finance University, Baoding, China

**Keywords:** language teaching, visual art, language learning performance, language satisfaction, curriculum and environment

## Abstract

Innovative technological products are present in students' environments. Information explosion and popularity are affecting their thoughts, and there is a large amount of information fuel in life through the Internet, television, movies, and advertisements. This phenomenon transforms reading from pure words into image. The current study was conducted using an experimental design model. A total of 188 ethnic minority students in the Hebei Province participated in the experimental study. The experimental group went through language and visual art integrated instruction, while the control group underwent traditional teaching. The experimental study lasted for 20 weeks (3 h per week). The research results showed that (1) spoken and written artistic conception was displayed through artistic expression. Ethnic minority students' differences in language skills and artistic expression were found to be the major factors that were effective in the production process. These aspects made the work richer and even more diversified. (2) After joining the classes and covering several lessons, ethnic minority students were found to be getting increasingly better in terms of integrating art vocabulary into their conversation. (3) Language and visual art integrated teaching input was a story content, which was received through “listening”, while the outputs were individual opinions through “speaking”. Their thoughts were presented through “painting”. The creation process operated in the brain and reflected differences in terms of their thinking skills, vocabulary organizational skills, language use, and creativity. Based on the results, this study is expected to improve the language proficiency of ethnic minority students, enhance their artistic culture, and pave the way for their understanding and attitude toward language and literature.

## Introduction

Technology development has brought about many drastic changes in the daily life of people, especially students, in the 21^st^ century. Innovative technological products have taken the place of book reading in students' life. Information explosion and popularity are influencing their thoughts, and there is a large amount of information fuel in life through the Internet, television, movies, and advertisements. This phenomenon transforms reading from pure language and text into images (Hadi et al., 2021). With a view to sparing time for nurturing these visual activities, the use of language by students is consequently gradually getting reduced in the process. When the communication function of a language is replaced by pictures, children gradually ignore the information delivery function of the language. Language exchange nowadays is mostly limited by language use. It has been discovered that ethnic minority students have an insufficient language learning approach. Their concern regarding language words and sentences is also limited, which leads to a larger gap between them and their peers in the classroom. Their use of ambiguous language is weaker than the language spoken by urban students. It has been discovered that such a condition is common owing to the boom in technological products. Strong and figurative visual images replace words and sentences or articles, which can be comprehended after careful thinking. Ethnic minority students lose patience to reach that mental state so that they can learn efficiently from written works. On the contrary, they expect a quick input of visual, auditory, and sensory stimulation. They are poor at using language skills both to interpret their ideas and to maintain interpersonal communication. This is because of the emotional release and comprehension frustration that act as obstacles in the personal development process of ethnic minority students. Considering the above situations, it is important to integrate language teaching into the art education curriculum to open a learning opportunity for both art and language for ethnic minority students. This ethnic student-friendly approach can enable students to explore the language and literature through arts while deepening the learning of art through the use of language and literature. As a result, a win–win situation is achieved in artistic expression ability and language ability (Yundayani et al., 2019). The sensory stimulation of art in terms of multiple cultures leads ethnic minority students to feel and experience abstract theories and use art as a mediator to connect knowledge, increase the depth of learning, and instill in them the spirit of integrity. These changes in life, as a result, enable ethnic students to establish a connection with experience, life, and society, which has multiple aspects. Moreover, innovative thinking has also been encouraged to focus on various issues in the environment (Mayer et al., 2020). This point of view perceives art as the tool and medium that connects language and literature and uses art as an instrument. Besides, the essential value of art should be taken into consideration. For this reason, subject-based art education, which includes aesthetic experience and life experience in the visual art language and literature integrated curriculum, is proposed in this study. In addition, this study discusses the effect of language and visual art integrated teaching on students' language learning performance and satisfaction. It should be indicated that making language and art a part of ethnic minority students' life experience is considered important. These aspects provide an opportunity to discuss the effect of language and visual art integrated teaching on ethnic minority students' language learning performance and satisfaction. It is expected that the results of this study can help improve the language proficiency of ethnic minority students and enhance their artistic culture. It is also expected that this study helps them to develop an understanding and inculcate a positive attitude toward language and literature. In addition, this study is expected to contribute to their knowledge and culture of art, as well as feelings and experiences in life.

## Literature review

Language is an instrument that provides communication and a comprehensive subject; therefore, an effective language teaching approach requires integrated design and application (Stezycka and Etherington, 2020). The current society presents an environment with cultural diversity. In this environment, the coexistence of multiethnic languages and cultures possesses a potential for both harmony and troubles. The current education pattern faces the issue of including diverse student groups, and the family members of students also contain individuals from different cultural backgrounds in their family group itself. Single language teaching can no longer meet the requirements of all students. The development and evolutionary process of language has enriched the words with a variety of meanings with historical backgrounds. Each word presents the development and change in symbolic, phonetic, and literal meanings. Words are also evolved from pictures, and the relationship between pictogram and character provides clues to this process (Svenningsson et al., 2022). Different languages and cultures have their unique brilliance that it is necessary to keep the respectful attitude to explore a distinct culture. Arts is a field based on multicultural sensory stimulation, enabling the students to experience abstract theories with actual feelings. Students should also consider arts as the media for linking knowledge to enhance learning depth, width, and integrity. As a result of these explicable reasons, arts are intricately connected with students' experience, life, and society they live in and deal with various issues in the entire life environment from multiple perspectives and innovative thinking (Sunra-La Sunra et al., 2020). Such a point of view takes arts as a tool and medium to link language. It is the instrumental value of language that takes care of the essential value of arts.

Gao et al. (2019) considered language and visual art integrated teaching as an improved teaching experience. Knowledge being integrated by instructors allowed students to completely grasp the basics of the curriculum. Applying an intradisciplinary or interdisciplinary curriculum enables the establishment of a connection between language and arts. In addition, it contributes to inform students about the two fields together and allows students to gain experience and find out the connections between them. Even though students cannot successfully find the connecting point at the time, they can accumulate adequate knowledge for self-awareness in the future. Preece and Marshall (2020) mentioned that, in addition to visual art, other subjects were covered in the language and visual art integrated instruction model. The art works and students' works are placed in the social, cultural, historical, and artistic aesthetic context. This is because the meaning of a work can be reinforced through the level of relationship with other art works, objects, artificial works, and events in other subjects. These events include various art-related fields such as learning art creation, art history, aesthetics, and art criticism. Besides, they attempted to affect and even generalize the events to other relevant subjects based on visual art education (Brown et al., 2020). Santos and Castro (2021) stated that language and visual art integrated teaching, along with different curriculum goals and through the adjustment of teaching content, can help achieve the learning outcome more effectively. The use of art instruction to support the other subjects in achieving subject-specific and cross-curricular learning objectives can explain the integration and recognition of issues in students' lives. Language and visual art integrated instruction, with an art-based integrated curriculum, uses art as a medium. In addition, art concept, aesthetic elements, and art resource were also used to mediate, activate, assist, and integrate learning in other fields to present language through paintings and to support the expression in paintings (Mayer et al., 2020). The word has a rich and unlimited meaning that gradually affects the students' learning performance (Rahmatika et al., 2021). Accordingly, the following hypothesis is proposed in this study.

**H1:** Language and visual art integrated instruction has significant effects on language satisfaction.

The contents and styles of language and literature application have become an instrument for recording the ideology. Language and literature, as a comprehensive subject, provide a tool that can stimulate thinking and deliver thoughts. They can also help students construct logic, practice personality education, transmit history, and improve communication through listening and reading. Therefore, integrated design and application are necessary for language and literature instruction. Art education emphasizes art perception to promote the perception of aesthetics in life, appreciate the beauty of art work, and develop artistic creation through awareness, as well as integrate aesthetics into daily life (Choe et al., 2019). Language and visual art are two different creation styles. After thousand years of accumulation, collection, change, and transformation, they are presented in various formats but have close relationships (Schmid et al., 2021). Johann and Bülow (2019) considered the art work as independent from the objective world and the subjective mind, so that it is a creation in the true sense. If it were simply the deviant form of an object, the spoken language could reveal the way to store information. People used spoken language to record anecdotes that were passed down from generation to generation and became stories that spread widely. Spoken language did not leave behind material products but was passed down through memory and served as a way for people to reflect (Noyan Erbaş et al., 2021). Image also provides the opportunity to interpret the connotation of the word and reveals the author's thoughts through symbols, metaphors, hints, colors, and compositions in the image, so visual communication is more shocking and effective than literature. Consequently, language teaching that integrates visual art can significantly affect students' satisfaction in language learning (Purwanto et al., 2020). Therefore, the following hypothesis is proposed in this study.

**H2**: Language and visual art integrated instruction has significant effects on language learning performance.

Su (2019) considered the preference for different behaviors, attitudes, and feelings toward learning activities as learning satisfaction. Students with higher learning satisfaction showed better learning outcomes, which therefore became the most important objective of differentiated learning. Copur-Gencturk and Thacker (2021) discussed the relationships between learning satisfaction and learning outcomes and found that learning satisfaction had a significant positive effect on learning outcomes. Shao et al. (2019) studied the relationships between learning satisfaction and learning outcomes of sports talent class students in elementary schools and found out moderately positive correlations between learning satisfaction and learning outcomes of the participants in Taipei City and New Taipei City. Kaliisa et al. (2019) investigated learning satisfaction and learning success of high school students in swimming classes and found significant positive correlations between learning satisfaction and learning outcomes of students in swimming classes. Preece (2019) used G6 as an example to investigate e-book learning outcomes and learning satisfaction and found out the remarkably positive correlations. Therefore, the following hypothesis is proposed in this study.

H3: Language satisfaction has significant positive effects on language learning performance.

## Methodology

### Measurement of research variable

#### Language satisfaction

Following the study of Wang et al. (2021), in this study, we considered two factors of learning satisfaction as teacher's instruction and curriculum and environment.

1. Teacher's instruction: Teachers' professional knowledge, ability to solve students' problems, preparation before lessons, teaching styles, teaching attitude, interaction with students, and understanding of students' individual needs can improve students' learning satisfaction.
2. Curriculum and environment: Learners' interests in the course content and their behaviors during the learning process improve their positive attitude.

#### Language learning performance

Following the study of Fang et al. (2022), factors in students' learning outcomes were proposed as follows.

1. Instructional factors: These include peer relationships, student–teacher interaction, classroom equipment, learning environment, family background, and community's cultural values.
2. Environmental factors: These factors include teaching style, teaching hour, curriculum design, material content, and teachers' organization and lecturing ability.

### Research object and sampling data

There are 55 different ethnic minorities in Hebei Province, China, with a population of 2.96 million. This constitutes about 4.27% of the total population in the province and is ranked ninth nationally. Native ethnic minorities include the Manchus, the Hui people, the Mongolians, and the Koreans. Ethnic minorities in the Hebei Province are distributed in urban and rural areas. Among them, 83% live in villages, while 17% of them live in cities. Several native ethnic minorities inhabit a certain region, for instance, Manchu reside mainly in Chengde, Qinhuangdao, Zunhua of Tangshan, and Yi County of Baoding. Similarly, Hui people reside mainly in villages and towns in Cangzhou and Langfang as well as in some larger towns in other cities. Most ethnic minorities inhabit in poor areas with severe natural conditions and low fertility of the soil. For instance, they live in Taihang Mountains, high and cold areas on a dam, and lower areas with alkali soil along Heilongjiang River. Ethnic minority students in China have worse language learning and artistic culture achievement compared to urban students. For this reason, the experimental design model with language and visual art integrated teaching for ethnic minority students was adopted in this study. It is expected to understand train ethnic minority students' language proficiency, improve their level of artistic culture, improve their understanding and feeling of language and literature, as well as their knowledge and culture of art.

A total of 188 ethnic minority students in Hebei Province participated in the experimental study. The experimental group went through language and visual art integrated instruction, while the control group maintained traditional teaching. The experimental study lasted 20 weeks (3 h per week). SPSS Software was used for data analysis. Factor analysis, reliability analysis, regression analysis, and analysis of variance were conducted to test the hypotheses.

### Research process

Creating a research gap and finding an appropriate sample is of extreme importance in the research process. According to the research gap, a literature review and analysis were conducted. Based on these theories, the design of the teaching unit was drafted, and the course contents were prepared for the teaching purpose. Besides, students' in-class work and creation processes were observed, recorded, and collected in the teaching activity. On the collected data, unit review and reflection were done after the end of each unit. This enabled teachers to revise the successive teaching activities. Students were given the questionnaire survey after the end of the course. With this, it was aimed to analyze the language satisfaction and the language learning performance of the students.

The teaching experiment was implemented for 20 weeks, from January to May 2022. The course descriptions and evaluation standards were explained in the first week. The language teaching integrated with visual arts was conducted as scheduled from the second week, and the general revision and the questionnaire survey were conducted in the last week. The teaching contents included “understanding the development process of impressionism and announcing the viewpoints of impressionist works”, “creating personal work based on the principles of impressionism”, “enhancing the learning effectiveness of art creation through language and improving self-confidence and creating positive experience”, “presenting the artistic conception or content of language with visual creation”, “observing the relations between affairs in daily life and oneself with visual performance”, “enhancing the ability to appreciate and comment personal and others' works through language ability”, and “enhancing the art and language performance through teaching activity for experience accumulation”. These contents were designed to match the teaching objectives. With a traditional teaching model, the course explanation and evaluation standards were also conducted in the first week. The course was conducted as mentioned in the schedule from the second week, and the general revision and questionnaire survey were implemented in the last week. The teaching content focuses on enhancing listening, speaking, reading, and writing skills in the field of language. As in traditional teaching, teachers followed the principles of didactic teaching.

### Data collection and analysis

The research data were collected with a questionnaire survey as well as the instructor's teaching practice through in-class video and tape recording and teacher's observation for the analysis.

Quantitative research, a structure-oriented measurement method for a specific phenomenon, considers that any individual, organizational, or social truth be defined and manipulated (Miller and Brewer, 2003). The researcher established the observed social phenomena or human behaviors' logic and presented with numbers to explore the relations through statistics. This enabled the social fact to be objectively and systematically studied to understand the cause-and-effect among variables (Outhwaite and Turner, 2007). With these features in mind, it was decided that the quantitative research conforms to the objectives of this study that is used for this study. Besides, the questionnaire survey results could be easily quantified. A questionnaire survey is a structured survey, with a fixed form of expression, questioning orders, and the answering method of the questions. Besides, it is a kind of text exchange. Accordingly, nobody, including the researchers or surveyors too, can interfere with the survey with subjective bias. Since it was reported that the statistical results can be quantified (Outhwaite and Turner, 2007), the quantitative research method was utilized for this study, and a questionnaire survey was used.

## Analysis results

### Reliability and validity analysis

The factor analysis was used and two factors, namely, “teacher's instruction” (eigenvalue = 2.561, α = 0.87) and “curriculum & environment” (eigenvalue = 2.192, α = 0.89), were extracted from the language satisfaction construct. The cumulative covariance explained was found as 75.262%.

The factor analysis for the language learning performance construct generated two factors: “instructional factors” (eigenvalue = 4.327, α = 0.93) and “environmental factors” (eigenvalue = 3.183, α = 0.95). The cumulative covariance was found to be 77.128%.

### Effects of language and visual art integrated instruction on language satisfaction and language learning performance

#### Analysis of variance of language visual art integrated instruction in language satisfaction

The ANOVA, which was conducted to discuss the difference of teaching model in language satisfaction (Table 1), showed significant differences between teaching model and teacher's instruction. Based on teachers' teaching, language teaching combined with visual arts (4.12) was found to have a higher score than the traditional teaching mode (3.34). Similarly, based on curriculum and environment, the integration of language teaching with visual arts (4.05) was found to have a higher score than the traditional teaching mode (3.51). Therefore, H1 was supported.

**Table 1:** Variance analysis of language and visual art integrated instruction in language satisfaction.

**Variable**		**F**	***P*-value**	**Scheffe *post-hoc***
Language and visual art integrated instruction	Teacher's instruction	17.534	0.000*	Language and visual art integrated instruction (4.12) > traditional teaching model (3.34)
	Curriculum and environment	22.893	0.000*	Language and visual art integrated instruction (4.05) > traditional teaching model (3.51)

* p < 0.05.

#### ANOVA of language and visual art integrated instruction in language learning performance

The ANOVA was implemented to discuss the difference between teaching model and language learning performance (Table 2). The results showed significant differences in terms of the teaching model in language learning performance. In the integration of language teaching with visual arts as compared to traditional teaching mode, language teaching with visual arts (4.23) was found to be higher on teaching factors than the traditional teaching mode (3.56). Similarly, in the integration of language teaching with visual arts as compared to traditional teaching mode, the integration of language teaching with visual arts (4.17) was found to be higher on environmental factors than the traditional teaching mode (3.48). In this respect, it can be stated that H2 was supported.

**Table 2:** Variance analysis of language and visual art integrated instruction in language learning performance.

**Variable**		**F**	***P*-value**	**Scheffe *post-hoc***
Language and visual art integrated instruction	Instructional factors	19.732	0.000*	Language and visual art integrated instruction (4.23) > traditional teaching model (3.56)
	Environmental factors	25.467	0.000*	Language and visual art integrated instruction (4.17) > traditional teaching model (3.48)

* p < 0.05.

### Correlation analysis of language satisfaction and language learning performance

#### Correlation analysis of language satisfaction and instructional factors

To test H3, a correlation analysis was conducted (Table 3). The results revealed significant positive effects of teacher's instruction (β = 2.175^**^) and curriculum and environment (β = 2.241^**^) on instructional factors.

**Table 3:** Analysis of language satisfaction to language learning performance.

**Dependent variable → **	**Language learning performance**			
**Independent variable↓**	**Instructional factors**		**Environmental factors**	
**Language satisfaction**	**B**	**P**	**β**	**P**
Teacher's instruction	2.175**	0.000	2.394**	0.000
Curriculum and environment	2.241**	0.000	2.481**	0.000
F	37.438	42.596
Significance	0.000***	0.000***
R2	0.266	0.334
Adjusted R2	0.247	0.326

* p < 0.05,

** p < 0.01,

*** p < 0.001. Data source: Self-organized in this study.

#### Correlation analysis of language satisfaction and environmental factors

To test H3, a correlation analysis was conducted (Table 3). The results showed significant positive effects of teacher's instruction (β = 2.394^**^) and curriculum and environment (β = 2.481^**^) on environmental factors. Therefore, H3 was supported.

## Discussion

The research results revealed that, by using language and visual art integrated instruction, we can guide students to discuss the concept of impressionism (Su and Foulger, 2019). Moreover, this approach considers ethnic minority students as the subjects of the curriculum and emphasizes individual natural development while improving the educational value of art activity. The instructors select topics for integrating into the curriculum from various perspectives in order to contribute to ethnic minority students' development in various dimensions during the participation in the course (Tseng et al., 2019). The research hypothesis, namely H1: Language and visual art integrated instruction has significant effects on language satisfaction, is supported, which conforms to the research results of Hasan et al. (2021), Rahmatika et al. (2021), and Santos and Castro (2021). The integrated curriculum systematically integrates language and visual art knowledge for ethnic minority students to enable them to completely grasp the content. The teaching process improves the ethnic minority students' language ability and deepens their knowledge of the culture of art (van Lancker and Parolin, 2020). The experimental instruction analyzes ethnic minority students' art and language performance during their participation in the language and visual art integrated course. The research hypothesis, namely H2: Language and visual art integrated instruction has significant effects on language learning performance, is supported, conforming to the research results of Purwanto et al. (2020), Noyan Erbaş et al. (2021), and Schmid et al. (2021). The instructor particularly paid attention to the extension and correlation of teaching in the curriculum design to ensure a connection between units. It promotes ethnic minority students' learning motivation using the familiar elements with the new content in the course considering their prior knowledge. Regarding the use of media and art vocabulary, language was integrated into ethnic minority students' daily used word files and their painting skills were also advanced through repeated practice (Verkasaloa et al., 2021). Language and literature activity was also integrated into the curriculum. The appreciation of language and story was inculcated in different ways to deliver the word meaning through artistic creation. Story and personal experience are connected with unlimited performance to apply to the instruction to observe ethnic minority students' learning and development in various dimensions. The research hypothesis, namely H3: Language satisfaction has significant positive effects on language learning performance, is supported, conforming to the research results of Kaliisa et al. (2019), Preece (2019), and Copur-Gencturk and Thacker (2021). It is evident that the course diversity of language teaching combining visual arts was favored by students. Different from monotonous basic subjects, students preferred free, flexible, and limitless creation courses. An integrated teaching of language and visual arts was favored by students, and it encourages students' willingness to learn both fields.

## Conclusion

The experimental result revealed that language and visual art integrated teaching (4.12) was found to be higher than the traditional teaching mode (3.34) on teachers' teaching, and language and visual arts' integration (4.05) was found to be higher than that taught by traditional teaching mode (3.51) on curriculum and environment. Therefore, H1 is supported: Language and visual art integrated instruction has significant effects on language satisfaction.

Language teaching combined with visual arts (4.23) was found to higher on teaching factors than the traditional teaching mode (3.56), and language and visual arts integration (4.17) was found to be higher on environmental factors than the traditional teaching mode (3.48). Therefore, H2 is supported: Language and visual art integrated instruction has significant effects on language learning performance.

The correlation analysis results of language satisfaction and teaching factors showed notable and positive effects of teacher's teaching (β = 2.175^**^) and curriculum and environment (β = 2.241^**^) on teaching factors. In addition, the correlation analysis results of language satisfaction and teaching factors showed significant and positive effects of teacher's teaching (β = 2.394^**^) and curriculum and environment (β = 2.481^**^) on environmental factors. The research hypothesis, namely H3 is, therefore, supported: Language satisfaction has significant positive effects on language learning performance.

The experiment revealed that language and visual art integrated teaching provides input through listening and reading, without the assistance of pictures. The composition of pictures was stimulated by language and comprehended, induced, and transformed into individually perceived concepts (Qiu et al., 2021). Ethnic minority students were trained in terms of concentration, quick thinking, ideation, memory, and emotional perception through listening to ensure proper judgment and analysis in the listening process. Words were transformed into specific images, feelings, and thoughts during reading comprehension. As a result, their thinking and imagination to comprehend the story content and integrate with artistic expression for the final objective of self-actualization was reached (Yang et al., 2020). The artistic concept of language and word was presented through an artistic expression. The differences in language proficiency and artistic expression of ethnic minority students are the factors of the production process. These aspects led the work to be richer and more diversified (Buttler, 2020). Teachers encourage ethnic minority students to deliver their personal opinions with art vocabulary and integrate art terminology into the language and discussion so that their individual presentation is contextual and full of affection. Teachers largely used classroom for demonstration, and ethnic minority students started to add terminology into the language. After showing their improvement in various lessons, ethnic minority students started to get better and better to integrate art vocabulary into language conversation. Language and visual art integrated teaching input was presented *via* the story content through “listening”. The output regarding their personal opinions was in the form of “speaking” and “painting”. Ethnic minority students absorbed external information with these three actions for internalization and organization. They interpreted the course content with their personal opinions and previous experience and transformed it into language and painting for output. The creation process was operated in the brain. Ethnic minority students were trained and was asked to observe the transformation and the reflected thinking skills, language organizational skills, language use skills, and creation skills' outcomes.

## Data availability statement

The original contributions presented in the study are included in the article/supplementary material, further inquiries can be directed to the corresponding author.

## Ethics statement

The present study was conducted in accordance with the recommendations of the Ethics Committee of the Minzu University of China, with written informed consent being obtained from all the participants. All the participants were asked to read and approve the ethical consent form before participating in the present study. The participants were also asked to follow the guidelines mentioned in the research form. The research protocol was approved by the Ethics Committee of the Minzu University of China.

## Author contributions

YZ performed the initial analyses and wrote the manuscript. SJ assisted in the data collection and data analysis. All authors revised and approved the submitted version of the manuscript.

## Conflict of interest

The authors declare that the research was conducted in the absence of any commercial or financial relationships that could be construed as a potential conflict of interest.

## Publisher's note

All claims expressed in this article are solely those of the authors and do not necessarily represent those of their affiliated organizations, or those of the publisher, the editors and the reviewers. Any product that may be evaluated in this article, or claim that may be made by its manufacturer, is not guaranteed or endorsed by the publisher.

## References

[B1] Brown T. Mann B. Ryder N. Subbiah M. Kaplan J. D. Dhariwal P.. (2020). Language models are few-shot learners. Adv. Neural. Inf. Processi. Syst. 3, 877–1901.

[B2] Buttler T. (2020). Disrupting my teaching practices: A teacher educator living as a contradiction. Stud. Teach. Educ. 16, 222–239. 10.1080/17425964.2020.1758654

[B3] Choe R. C. Scuric Z. Eshkol E. Cruser S. Arndt A. Cox R.. (2019). Student satisfaction and learning outcomes in asynchronous online lecture videos. CBE Life Sci. Educ. 18, 1–14. 10.1187/cbe.18-08-0171 31675279 PMC6829069

[B4] Copur-Gencturk Y. Thacker I. (2021). A comparison of perceived and observed learning from professional development: relationships among self-reports, direct assessments, and teacher characteristics. J. Teach. Educ. 72, 138–151. 10.1177/0022487119899101

[B5] Fang T. Wang L. Y. Lin T. B. Huang C. K. (2022). To stay or leave: a multiple-case study of the retention of native English-speaking teachers in Taiwan. Asia Pacific. Educ. Rev. 23, 325–340. 10.1007/s12564-022-09756-7

[B6] Gao J. He D. Tan X. Qin T. Wang L. Liu T. Y. (2019). Representation degeneration problem in training natural language generation models. arXiv preprint arXiv:1907.12009.

[B7] Hadi M. S. Izzah L. Paulia Q. (2021). Teaching writing through canva application. J. Lang. Teach. Res 9, 228–235. 10.33394/jollt.v9i2.3533

[B8] Hasan S. I. Yee A. Rinaldi A. Azham A. A. Hairi F. M. Nordin A. S. (2021) Prevalence of common mental health issues among migrant workers: A systematic review meta-analysis. PLoS ONE. 16, e0260221. 10.1371/journal.pone.0260221 34855800 PMC8638981

[B9] Johann M. Bülow L. (2019). One does not simply create a meme: Conditions for the diffusion of Internet memes. J. Int. Commun 13, 23.

[B10] Kaliisa R. Palmer E. Miller J. (2019). Mobile learning in higher education: A comparative analysis of developed and developing country contexts. Br. J. Educ. Technol. 50, 546–561. 10.1111/bjet.12583

[B11] Mayer R. E. Fiorella L. Stull A. (2020). Five ways to increase the effectiveness of instructional video. Educ. Technol. Res. Dev. 68, 837–852. 10.1007/s11423-020-09749-6

[B12] Miller R. L. Brewer J. D. (2003). A-Z of Social Research. London: SAGE Publications Ltd; Longman. 10.4135/9780857020024

[B13] Noyan Erbaş A. Özcebe E. Cak Esen T. (2021). Investigation of the effect of Hanen's “More Than Words” on parental self-efficacy, emotional states, perceived social support, and on communication skills of children with ASD. Logoped Phoniatr. Vocol. 46, 17–27. 10.1080/14015439.2020.1717601 32003252

[B14] Outhwaite W. Turner S. P. (2007). The SAGE Handbook of Social Science Methodology. London: SAGE Publications Ltd; Longman. 10.4135/9781848607958

[B15] Preece S. (2019). Postgraduate students as plurilingual social actors in UK higher education. Lang. Cult. Curric. 33, 126–141. 10.1080/07908318.2019.1676767

[B16] Preece S. Marshall S. (2020). Plurilingualism, teaching and learning, and Anglophone higher education: An introduction Anglophone universities and linguistic diversity. Lang. Cult. Curric. 33, 117–125. 10.1080/07908318.2020.1723931

[B17] Purwanto A. Asbari M. Fahlevi M. Mufid A. Agistiawati E. Cahyono Y.. (2020). Impact of work from home (WFH) on Indonesian teachers performance during the COVID-19 pandemic : an exploratory study. Int. J. Adv. Sci. Technol. 29, 6235–6244.

[B18] Qiu Q. Xie Z. Xiong Y. Zhou F. (2021). Belief change before and after the teaching practicum among Chinese pre-service ELT teachers. Sage Open 11, 21582440211004934. 10.1177/21582440211004934

[B19] Rahmatika R. Yusuf M. Agung L. (2021). The effectiveness of youtube as an online learning media. J. Educ. Technol. 5, 152. 10.23887/jet.v5i1.33628

[B20] Santos J. M. Castro R. D. R. (2021). Technological Pedagogical content knowledge (TPACK) in action: Application of learning in the classroom by preservice teachers (PST). Soc. Sci. Human Open 3, 100110. 10.1016/j.ssaho.2021.100110

[B21] Schmid M. Brianza E. Petko D. (2021). Self-reported technological pedagogical content knowledge (TPACK) of pre-service teachers in relation to digital technology use in lesson plans. Comput. Hum. Behav. 115, 106586. 10.1016/j.chb.2020.106586

[B22] Shao K. Pekrun R. Nicholson L. J. (2019). Emotions in classroom language learning: What can we learn from achievement emotion research? System 86, 102121. 10.1016/j.system.2019.102121

[B23] Stezycka P. E. Etherington S. (2020). Teacher motivation: a study of polish secondary school EFL teachers. TESL-EJ 24, 1–28.

[B24] Su F. L. (2019). Wandering at the crossroad between language maintenance and language shift-a survey on language attitudes toward taigi among 3rd - 9th graders in Taiwan. J. Taiwan Lang. Lit. 14, 81–119. 10.6710/JTLL.201904_14(1).0003

[B25] Su M. Foulger T. S. (2019). We Aren't There Yet: A Progression of literature on TPACK measures to assess technology integration. In: Society for Information Technology & Teacher Education International Conference. p. 2534–2542.

[B26] Sunra-La Sunra L. Haryanto H. Nur S. (2020). Teachers' Reflective Practice and Challenges in an Indonesian EFL Secondary School Classroom. Int. J. Lang. Educ. 4, 289–300. 10.26858/ijole.v4i2.13893

[B27] Svenningsson J. Höst G. Hultén M. Hallström J. (2022). Students' attitudes toward technology: Exploring the relationship among affective, cognitive and behavioral components of the attitude construct. Int. J. Technol. Des. Educ. 32, 1531–1551. 10.1007/s10798-021-09657-7

[B28] Tseng H. C. Chen B. Chang T. H. Sung Y. T. (2019). Integrating LSA-based hierarchical conceptual space and machine learning methods for leveling the readability of domain-specific texts. Nat. Lang. Eng. 25, 331–361. 10.1017/S1351324919000093

[B29] van Lancker W. Parolin Z. (2020). COVID-19, school closures, and child poverty: a social crisis in the making. Lancet Public Health 5, e243–e244. 10.1016/S2468-2667(20)30084-0 32275858 PMC7141480

[B30] Verkasaloa E. J. Silvenb M. Lehtioa I. Eggersa C. K. (2021). Speech disfluencies in typically developing Finnish-speaking children – preliminary results. Clin. Linguist Phon. 35, 707–726. 10.1080/02699206.2020.1818287 32993385

[B31] Wang Y. Cui L. Zhang Y. (2021). Improving skip-gram embeddings using BERT. IEEE/ACM Trans. Audio, Speech Lang. Process. 29, 1318–1328. 10.1109/TASLP.2021.306520133010425

[B32] Yang C. Hawwash D. De Baets B. Bouwman J. Lachat C. (2020). Perspective: towards automated tracking of content and evidence appraisal of nutrition research. Adv. Nutr. 11, 1079–1088. 10.1093/advances/nmaa057 32504536 PMC7490154

[B33] Yundayani A. Susilawati S. Chairunnisa C. (2019). Investigating the effect of canva on students' writing skills. English Rev. J. Eng. Edu. 7, 169–176. 10.25134/erjee.v7i2.1800

